# Predictive Analysis of Extubation Failure in the Paediatric Intensive Care Unit in Bloemfontein, South Africa

**DOI:** 10.3390/reports9020169

**Published:** 2026-05-28

**Authors:** Mbaya Buankuna, Joseph B. Sempa, Olive P. Khaliq, Michael A. Pienaar

**Affiliations:** 1 Department of Paediatrics and Child Health, School of Clinical Medicine, Faculty of Health Sciences, University of the Free State, Bloemfontein 9301, Free State, South Africa; khaliqop@ufs.ac.za (O.P.K.); pienaarma1@ufs.ac.za (M.A.P.); 2 Department of Biostatistics, School of Clinical Medicine, Faculty of Health Sciences, University of the Free State, Bloemfontein 9301, Free State, South Africa; sempajb@ufs.ac.za

**Keywords:** extubation failure, paediatrics, intensive care, mechanical ventilation

## Abstract

**Background:** Extubation failure (EF) is a significant complication, and it is associated with increased mortality, prolonged hospital stays and extended mechanical ventilation (MV). Determining reliable predictors of EF could improve the clinical decision-making and outcomes. **Objectives:** Determine the outcomes and predictors of EF in a paediatric intensive care unit (PICU) and develop predictive models using machine learning algorithms. **Methods:** A retrospective cohort study (n = 824) was conducted in two PICUs in participants who underwent planned extubation (January 2018–December 2022). Demographic characteristics, clinical parameters, ventilator setting, laboratory findings and extubation outcomes were collected. Univariate and multivariate analysis were performed to identify significant predictors of EF. Six machine learning algorithms—Logistic Regression (LR), Artificial Neural Network (ANN), Extreme Gradient Boosting (XGBoost), Random Forest (RF), Support Vector Machine (SVM) and Decision Tree (DT)—were developed and validated for prediction of EF. **Results:** The overall EF rate was 231 (28%). Multivariate analysis identified a mechanical ventilation for a duration of 3 days or more (aOR 4.49, 95% CI 3.24–6.57, *p* < 0.001), use of neuromuscular blockade (aOR 1.32, 95% CI 1.07–1.63, *p* = 0.009), and administration of vasopressors (aOR 1.57, 95% CI 1.24–2.01, *p* < 0.001) as significant independent predictors of EF. The ANN and LR models demonstrated the highest performance with AUCROC of 0.87 ± 0.04 and 0.86 ± 0.02, respectively. **Conclusions:** Extubation failure was common in our setting (28%) compared to other studies. Days of ventilation, undernutrition, use of neuromuscular blockade, use of vasopressors or inotropes and CNS comorbidity were associated with EF. The main cause of EF was upper airway obstruction.

## 1. Introduction

Mechanical ventilation (MV) has decreased mortality rates in critically ill patients [1,2]. In the United States, approximately 800,000 patients are mechanically ventilated annually [3]. Despite its benefits, mechanical ventilation is associated with significant complications. These include pneumothorax, ventilator-associated pneumonia, ventilator-associated lung injury, and extubation failure (EF) [3,4].

Invasive mechanical ventilation should be terminated as soon as the patient is able to maintain spontaneous breathing and reasonable gas exchange [5,6,7]. The process of extubation should be undertaken as soon as the patient can sustain independent respiration [8,9]. Even after a favourable spontaneous breathing trial (SBT), approximately 15% of patients will require re-intubation [6,10]. There are currently no reliable tests to predict extubation readiness in children; the decision to extubate remains largely at the discretion of treating clinicians [5,11]. Liu et al. found that a forecasting model using medication response and patient characteristics better predicted extubation readiness [12], while Moore et al. highlighted the importance of clinical assessment [13].

Extubation failure, defined as the need for reintubation within 48 h after planned extubation, increases the risk of prolonged ventilation, mortality, and prolonged hospital stay [10]. In adult studies, predictors of extubation failure have been investigated, but data in the paediatric population are limited. Zhao et al. identified 19 features out of 89 that can predict extubation failure: age, body mass index (BMI), mean arterial pressure (MAP), respiratory rate, tidal volume, mean airway pressure, spontaneous breathing trials (SBT) and urine output are among the 19 mentioned features [10].

Globally, several models have been developed using machine learning (ML) to predict extubation outcomes, aiming to minimise the risk of re-intubation and its associated complications [5,7,14]. Rooney et al. showed that physiologic variables, such as peripheral oxygen saturation extremes, dynamic compliance, central venous pressure, and heart/respiratory rates, emerged as key predictors of EF using RF in a paediatric cardiac ICU [15]. In this context, the use of ML models may help predict the outcome of extubation by supporting the physician in the decision-making process.

Both Universitas Academic Hospital and Pelonomi Tertiary Hospital in Bloemfontein, South Africa, offer invasive mechanical ventilation in their respective paediatric intensive care units (PICUs). The physician’s judgement guides the decision to extubate in these two centres.

There is a need to develop an acceptable tool to predict extubation outcomes. ML models could be used to achieve this task using clinical and ventilator setting features. The aim will be to develop and validate a model of EF in mechanically ventilated patients in PICU. Machine learning is a subdiscipline of artificial intelligence focused on specific tasks: in this case, prediction [16]. Machine learning is generally divided into two categories: supervised and unsupervised learning [11,14].

Supervised machine learning uses a certain number of algorithms, of which the most common are Decision Tree (DT), Random Forest (RF), Gradient Boosting (GB), Light Gradient Boosting Model (LightGBM), Extreme Gradient Boosting (XGBoost), Logistic Regression (LR), Support Vector Machine (SVM), CatBoost, Adaboost, Multilayer Perceptron (MLP), K-Nearest Neighbour (KNN), NaiveBayes [10].

In an unsupervised machine learning model, patterns are inferred from unlabelled data. The objective is to identify the structure and patterns in the input data.

For this study, the focus will be on the supervised machine learning model using the following algorithms: Decision Tree (DT), Artificial Neural Network (ANN), Extreme Gradient Boosting (XGBoost), Random Forest (RF), Support Vector Machine (SVM), and Logistic Regression (LR).

In this study, the researcher aimed to investigate risk factors for EF and to develop supervised ML models for predicting EF in mechanically ventilated children.

## 2. Materials and Methods

### 2.1. Study Design

This is a retrospective cohort study conducted in two paediatric intensive care units (PICUs) in Bloemfontein, Free State Province, South Africa. The study was also a predictive modelling research design, using machine learning techniques. Data were collected from January 2018 to December 2022.

### 2.2. Study Site

The study was conducted in two paediatric intensive care units in Bloemfontein. Pelonomi Tertiary Hospital and Universitas Academic Hospital are both major healthcare facilities in the Free State province. Pelonomi Tertiary Hospital is a teaching hospital with a five-bed PICU, while Universitas Academic Hospital is a quaternary teaching hospital with a five-bed PICU, offering specialist and subspecialist paediatric care.

### 2.3. Study Population

The study included all patients under 13 years of age who were admitted to one of the PICUs and required invasive mechanical ventilation during the study period. Prolonged ventilation was defined as mechanical ventilation for more than 14 days, and difficult intubation as more than 2 unsuccessful attempts at direct laryngoscopy. Participants were eligible for inclusion in the study if they underwent a first time attempt of planned extubation. The exclusion criteria were as follows:

‑ Patients who self-extubated.
‑ Patients who were deemed for withdrawal of treatment.
‑ Patients with congenital upper airway obstruction.
‑ Patients extubated from high frequency oscillatory ventilation (HFOV).
‑ Patients with incomplete medical records.
‑ Patients who died while still on the ventilator.

### 2.4. Outcomes and Predictors of Extubation Failure

The primary outcome of interest was extubation failure, defined as the need for reintubation within 48 h following a planned extubation.

### 2.5. Data Collection

Data were retrospectively collected from patient medical records, including admission registers, the Paediatric Index of Mortality 3 (PIM 3) database, and Meditech electronic health records. A standardised data collection form was used to ensure consistency. The following variables were collected:

‑ Demographic data: Age, sex, referral source, weight, height and nutritional status (classified according to WHO criteria).
‑ Clinical data: PIM-3 mortality risk, diagnosis, comorbidities, neuromuscular blockade (NMB), vasoactive medication, and details of mechanical ventilation (mode, duration, PIP, PEEP, respiratory rate, FiO2).
‑ Extubation details: Timing of extubation, reasons for EF, and outcomes.
‑ Laboratory data: Relevant haematologic and biochemical markers, including blood gas and septic marker (procalcitonin or C-reactive protein).

All information was captured on a spreadsheet using REDCap.

Data were kept confidential by using a coding system. Only the researcher and the supervisor had access to the protected database. All patients included in the study were de-identified in compliance with ethical standards and institutional guidelines.

### 2.6. Statistical Analysis

Data collected were entered into Redcap and analysed using R programming, version 4.4.3 (R foundation for statistical computing, Vienna, Austria) and descriptive statistics were used to summarise demographic and clinical characteristics. Categorical variables were presented as frequencies and percentages, while continuous variables were summarised using medians and interquartile ranges (IQRs) or means and standard deviations (SD) as appropriate. Univariate analysis was performed using chi-square or Fisher’s exact tests for categorical variables and Mann–Whitney U or *t*-tests for continuous variables.

### 2.7. Model Development

Features were selected using Elastic Net regression, which combines the L1 (LASSO) and L2 (ridge) penalties to enable both variable selection and coefficient shrinkage [17] (see list of selected variables in Table 1). Elastic Net further addresses multicollinearity by stabilising coefficient estimates through the L2 penalty, which allows correlated predictors to be handled jointly, while the L1 component encourages sparsity by shrinking less informative predictors toward zero.

Multivariate Logistic Regression was used to identify independent predictors of extubation failure, with results presented as adjusted odds ratios (aORs) and 95% confidence intervals (CIs). A *p*-value of less than 0.05 (*p* < 0.05) was considered significant.

Models were developed in the Jupyter Notebooks (v 7.4.5) environment using Python 3. Packages used included Pandas, Numpy, Matplotlib, Scikit-Learn, Scikit Optimizer, Keras, Scikeras, Tensorflow, Scipy, and StatKit [18,19,20]. Missing data in the predictors was imputed using multiple imputation by chained equations [21]. Data was pre-processed by encoding categorical variables and scaling continuous variables to a scale of [0:1]. Class imbalance was identified in the data (28% positive class). This was addressed within training using balanced class weights for all models. In the ANN model, binary cross-entropy was used as the loss function and output layer bias was initialised as log(positive/negative) [22]. Further hyperparameters were tuned with an AUPRC goal, using Bayesian optimisation using one train–test split with a different random seed to the cross-validation. The full set of hyperparameters are presented in the Supplementary Table S1 (Annexure 1). Imbalanced data was further considered in model evaluation by consideration of the precision–recall curve. Models were then trained and validated within 5-fold stratified cross-validation.

### 2.8. Ethical Considerations

The study protocol was reviewed and approved by the Health Sciences Research Ethics Committee (HSREC) of the University of the Free State (UFS-HSD2023/1265/2609) and authorisation from the National Research Health database (NHRD) (FS 202308_022) was obtained. Given the retrospective nature of the study, informed consent was waived.

## 3. Results

**Figure 1:**
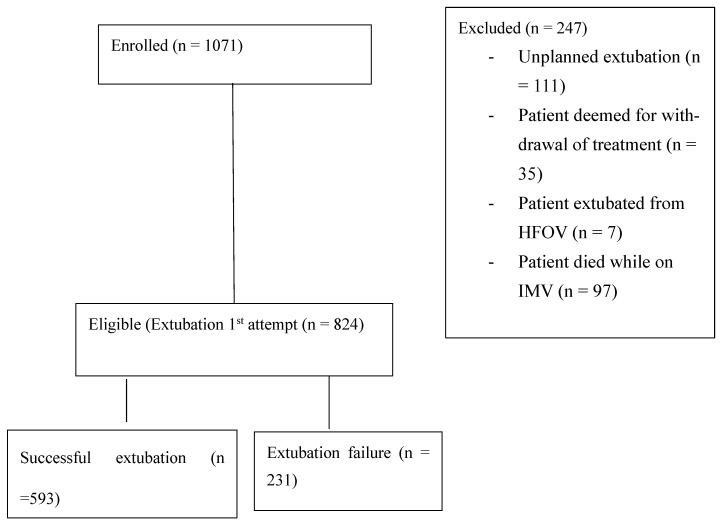
Schematic of patients enrolled in the study.

The study included 824 participants who underwent planned extubation in two PICUs in Bloemfontein, South Africa as shown in the schematic flow diagram (Figure 1). The median age of the participants was 9 months (IQR 2–48 months), with 59.3% of the participants being male. A total of 231 participants (28%) experienced extubation failure (Table 2). Patients referred from another facility were more prevalent in the failed extubation group (*p* < 0.001).

Most (80.0%) participants had normal nutritional status. Undernutrition was significantly associated with extubation failure (*p* = 0.029) (Table 2).

The presence of central nervous system comorbidity demonstrated a significant difference between the two groups (*p* = 0.022), while patients without comorbidities were extubated successfully, with a significant difference (*p* < 0.001) (Table 2).

The median days of ventilation were significantly higher in the extubation failure group than in the successful extubation group, at 3 days (IQR 3-3) and 2 days (IQR 2-3), respectively (*p* < 0.001) (Table 2). Upper airway obstruction was the most common reason for extubation failure (22.5%), followed by increased work of breathing (19.8%) and severe apnoea (3.0%), while sepsis and septic shock together accounted for only 1.5% (Table 2).

Multivariate Logistic Regression analysis was used to adjust for potential confounders. The analysis confirmed that a longer duration of mechanical ventilation (aOR 22.6, 95% CI 11.4–50.6, *p* < 0.001), use of neuromuscular blockade (aOR 3.73, 95% CI 1.75–8.39, *p* < 0.001) and need for vasoactive or inotropes during ventilation (aOR 2.06, 95% CI 1.26–3.37, *p* = 0.004) were independent predictors of extubation failure (Table 1).

**Figure 2:**
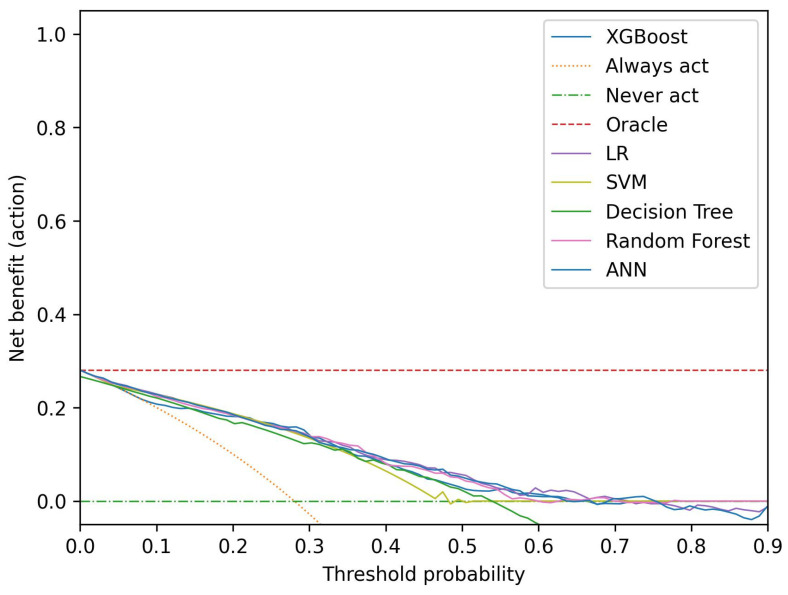
Decision curve analysis.

The decision curve analysis shows that all predictive models provide a positive clinical net benefit across a broader range of threshold probabilities than the “treat-all” and “treat-none” strategies. XGBoost, Logistic Regression, Random Forest, and ANN showed comparable performance and maintained clinical utility up to a threshold probability of approximately 0.60–0.65. In this range, the models demonstrate strong clinical utility in decision-making (Figure 2).

### 3.1. Prediction Model Performance

The study evaluated the predictive performance of six machine learning models: LR, ANN, XGBoost, RF, SVM, and DT. The models used in this study provide several important insights into the predictive performance of various machine learning and statistical methods for extubation failure.

### 3.2. Performance Metrics: AUCROC and AUCPRC

Logistic Regression (LR) and Artificial Neural Network (ANN) models demonstrated the highest AUCROC values (0.84 ± 0.02 for LR and 0.84 ± 0.02 for ANN), indicating strong discriminative ability. LR also had the highest AUCPRC value (0.65 ± 0.08), suggesting better performance in the minority class. ANN performed well with AUCPRC (0.64 ± 0.09). Extreme Gradient Boosting (XGBoost) also performed well, with AUCROC of 0.83 ± 0.02 and AUCPRC of 0.63 ± 0.05, making XGBoost an acceptable choice for prediction modelling. The relatively modest AUPRC values suggest moderate positive performance.

### 3.3. Calibration Metrics

**Figure 3:**
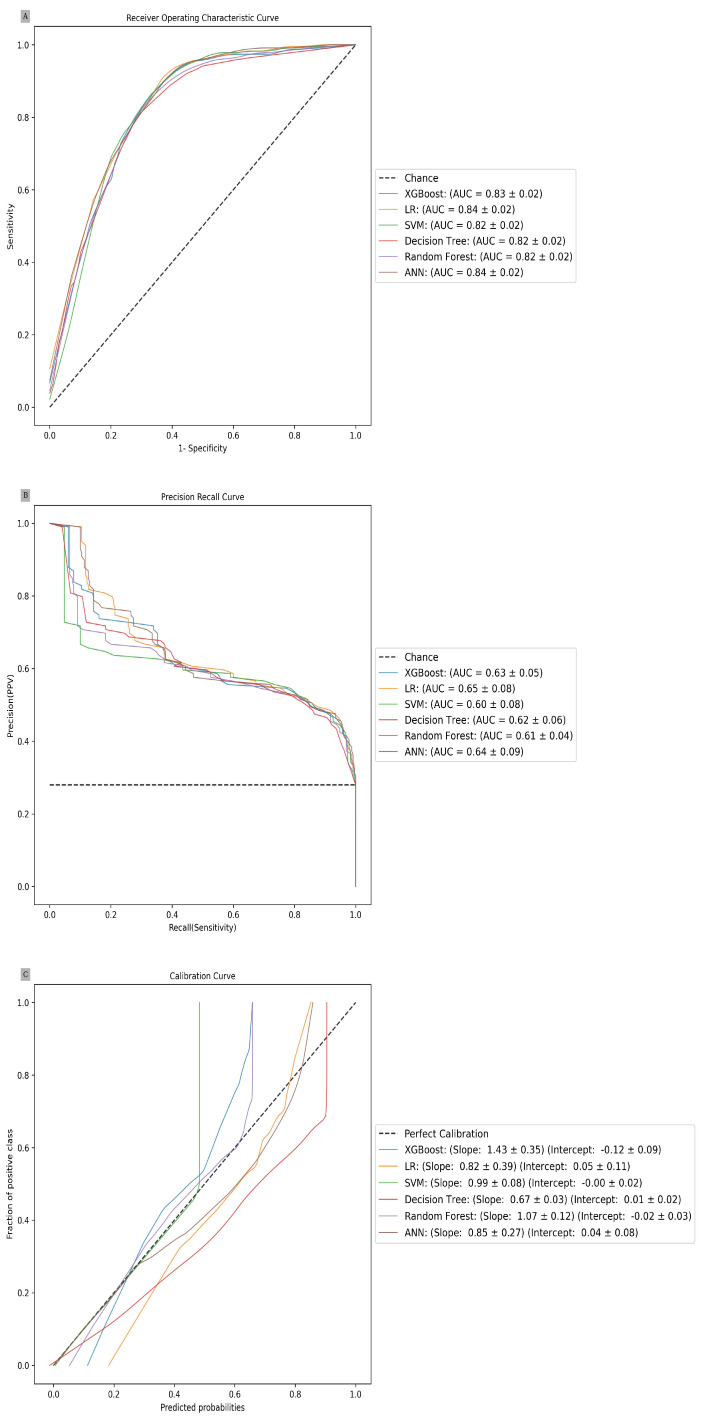
Model training and validation. (**A**): Receiver Operating Characteristic Curve (ROC): all models performed similarly with AUC values around 0.83–0.84, indicating good discrimination ability. (**B**): Precision–Recall Curve (PRC): Logistic Regression and XGBoost slightly outperform other models while all models converge toward lower precision at high recall. (**C**): Calibration Curve: most models show reasonable calibration with slopes close to 1 and small intercepts.

The calibration slope and intercept provide insights into how well the predicted probabilities align with the actual outcomes. LR had a calibration slope of 0.82 ± 0.39 and an intercept of—0.05 ± 0.11, suggesting the calibration is close to the perfect calibration line. ANN with a calibration slope of 0.85 ± 0.27 and intercept of—0.04 ± 0.08, and SVM with a slope of 0.99 ± 0.08, intercept—0.00 ± 0.02, also show good calibration. DT exhibit poor calibration (Figure 3).

### 3.4. Model Selection

Model selection should consider discrimination, calibration, clinical interpretability, and ease of implementation. Although all machine learning models demonstrate comparable discrimination performance, Logistic Regression emerged as the most balanced model due to its combination of strong discrimination, superior precision–recall performance, acceptable calibration, and high interpretability, while XGBoost and ANN demonstrate similar AUROC values but with less consistent calibration (Figure 3).

## 4. Discussion

The study identifies several factors associated with extubation failure in the paediatric intensive care unit. The results provide insights into the predictive variables and possible areas for further intervention.

### 4.1. Demographic and Clinical Characteristics

The study found that the EF rate in the two PICUs in Bloemfontein, South Africa, was 28%, which is higher than the rates reported in some previous studies. Newth et al. report an EF rate of 2 to 20% [11]; Kilba reports 11.4% [2]; and the study by Magose et al. reports an EF of 16.7% [23]. In their review article, Egbuta et al. found that the rate of EF ranged from 2.7% to 30% [8]. The discrepancy may be attributed to differences in patient characteristics, comorbidities, clinical practices, sedation protocols, and resource limitations in the study setting, such as staff shortages among nurses and doctors.

The median age of the study population was 9 months (IQR 2–48). This study did not demonstrate any association between age and EF. Still, Khemani et al. found that younger age was associated with higher EF rates, particularly in patients under 1 year old [24]. Kilba et al. found that the median age of patients who failed extubation was 3.1 months, but this factor was not a significant risk factor [2]. Carvalho et al. reported that younger age is associated with an increased risk of extubation failure, especially in the neonatal period, most likely due to respiratory system immaturity [25].

Undernutrition was significantly associated with EF (*p* = 0.0029). Grippa et al. highlighted that malnutrition significantly prolongs mechanical ventilation [26]. Mehta et al. pointed out the critical role of nutritional status in PICU outcomes, noting that malnutrition delays recovery and increases morbidity [27].

### 4.2. Predictors of Extubation Failure (EF)

Our findings indicate that prolonged mechanical ventilation (duration of MV > 14 days), the use of neuromuscular blockade, and the administration of vasopressors are significant predictors of EF. These results align with prior research on prolonged mechanical ventilation, which is a critical risk factor for respiratory muscle fatigue and the development of ventilation-associated complications [24,25]. The association between neuromuscular blockade and EF is also supported in the literature [9,24], likely due to the potential for prolonged muscle weakness following extended use of paralytic agents. Similarly, the use of vasopressors, which may indicate haemodynamic instability, has been linked to poorer extubation outcomes in our study. There is no consensus on the role of vasopressors as risk factors for extubation failure. Previous studies did not find an association between vasopressor use and EF [2,28].

### 4.3. Causes of Extubation Failure

The main reasons for extubation failure were upper airway obstruction (UAO), increased work of breathing and severe apnoea. Khemani et al. reported that respiratory complications were strongly associated with EF [24]. According to a previous report, UAO and inadequate respiratory effort were the primary causes of extubation failure [29,30]. Similarly, Simonassi et al. identified upper airway obstruction as a significant contributor to EF [31]. These findings underscore the importance of thorough respiratory monitoring and a comprehensive extubation-readiness assessment for all patients undergoing MV. Several factors may contribute to extubation failure, including difficult intubation, underlying neurological conditions, or a history of previous intubations. Abu-Sultanah et al. suggest using dexamethasone 6 h prior to extubation for children at high risk of post-extubation UAO. Still, the benefit of decreasing EF due to UAO was unclear [32].

### 4.4. Indications for Admission to PICU

In our study, respiratory diseases were the primary reason for admission in 43% of cases. The indications for ICU admission might vary between the two centres, Pelonomi and Universitas. Similarly, Carvalho et al. reported that respiratory pathologies and sepsis were common reasons for intubation and mechanical ventilation [25].

### 4.5. Comorbidities

Central nervous system (CNS) disease significantly increases the risk of EF (*p* = 0.018), whereas the absence of comorbidities appears to be protective against extubation failure. Simonassi et al. also found that patients with CNS diseases face a heightened risk of EF [31]. Given these findings, it is essential to implement thorough assessments and take all necessary precautions before extubating patients with CNS pathology.

### 4.6. Machine Learning Models

The study employed six different machine learning models to predict extubation failure, with Artificial Neural Network (ANN) and Logistic Regression (LR) models demonstrating the highest discrimination. The strong performance of these models suggests that machine learning can be a valuable tool in the PICU setting, providing clinicians with an evidence-based approach to assess extubation readiness [33,34]. LR, specifically, showed superior performance, indicating its potential for high sensitivity and specificity in predicting extubation outcomes. These findings are consistent with other studies that have applied machine learning to predict clinical outcomes in critical care [33]. However, while machine learning models offer promising predictive capabilities, it is essential to recognise that these models require extensive validation before they can be reliably integrated into clinical practice. The slight overfitting observed in the XGBoost model, as seen by its calibration metrics, underscores the need for careful model selection and tuning to avoid overly optimistic predictions [33].

### 4.7. Clinical Implications

Identifying key risk factors for extubation failure has important clinical implications. By incorporating machine learning models into clinical decision-making, PICU teams can better stratify patients by risk of extubation failure and tailor interventions accordingly. For instance, patients identified as high risk could benefit from aggressive respiratory support, closer monitoring, or extended weaning protocols. Recognising the role of factors such as undernutrition and central nervous system pathology in extubation outcomes highlights the need for a multidisciplinary approach to patient care, including nutritional support and neurological evaluation.

The implementation of such models in practice requires further investigation but can be considered at this stage. As these are risk-predictive models, a range of threshold probabilities [0.0 to 1.0] would provide grounds for some intervention. As false classifications are inherent to imperfect models, their nature becomes relevant to decision-making. As the threshold increases from zero, the number of false negative classifications will increase, while the number of false positive classifications will decrease. The consideration of how this threshold is set is thus dependent on the consequences of false negatives (in this case, extubation failure), the consequences of false positives (delays in extubation or other interventions), and the tolerance of the treating physician, patient, or patient’s proxy for these risks [35,36]. We propose possible implementations in decision support that could include risk-adjusted approaches to the timing of SBT (avoiding futile, premature SBT, which may cause patient discomfort or compromise respiratory function) or identifying patients who should undergo prolonged SBT or other evaluation, such as upper airway evaluation (e.g., leak testing or fibreoptic laryngoscopy). These interventions are relatively low-risk, and roleplayers may accept a lower threshold. On the contrary, decisions such as tracheostomy placement would likely require stronger evidence and higher thresholds. Notably, net benefit decreased to 0.0 for all models at threshold probability >0.8. Clinical translation of these models into practice will require larger, more robust training and validation data, as well as consultation and consensus-building processes for practice.

## 5. Conclusions

The extubation failure rate was higher in our setting (28%) than in other studies. Days of ventilation, undernutrition, neuromuscular blockade, vasopressors or inotropes, and CNS comorbidity were associated with extubation failure in our study. The main cause of extubation failure identified in the study was upper airway obstruction. While the models showed discriminative ability, the small sample size poses a risk of overfitting, and these findings provide a proof of concept, rather than models ready for clinical translation.

## 6. Limitations

1. The retrospective design of this study may introduce biases for data recording and data availability.
2. Most respiratory parameters and ventilator settings were not included in the analysis.
3. The duration and types of neuromuscular blockades and vasopressors/inotropes were not considered in this study.
4. Model calibration was moderate at best. Increased data size may be required to improve this metric.
5. The dataset was too small for predictive modelling. While cross-validation was used in model evaluation, the validation of this model is limited. As such, the generalizability of these models is uncertain, and their translational readiness is low. The small sample size further increases the risk of overfitting at various levels; as such, retraining on a larger data set and further validation, particularly on external data, are required before progressing to studies of their clinical use. What is demonstrated, however, is that the features studied are informative and that discriminative models can be developed to predict extubation failure. This publication also demonstrates that interpretable algorithms such as LR, DT and RF offer similar discrimination while providing explainable predictions for this application.

What the Study Adds: Extubation failure has been a more challenging step of ventilator liberation. This study shows that a machine learning model can help overcome these difficulties. The authors have demonstrated that mechanical ventilation for a duration of 3 days or more, use of neuromuscular blockade and vasopressor/inotropes are the main contributors to extubation failure, using a Logistic Regression model and an Artificial Neural Network.

Implications of the Findings: The identification of key risk factors for extubation failure helps to personalise extubation from mechanical ventilation and will contribute to establishing a protocol for extubation in PICU. This study will encourage researchers to undertake a prospective study and to deploy the current findings for validation.

Recommendations: Conduct a prospective study to validate these findings and seek external validation. Explore interventions directed at the risk factors identified in this study.

## Figures and Tables

**Table 1:** Multivariate logistic analysis of causes of extubation failure.

Characteristic	aOR	95% CI	*p*-Value
**Days of ventilation**	22.6	11.4–50.6	<0.001
**PIM-3 score**	4.44	0.78–25.2	0.092
**Vasopressors/inotropes use**	2.06	1.26–3.37	0.004
**Neuromuscular blockade use**	3.73	1.75–8.39	<0.001
**Corticosteroids pre-extubation**	1.64	0.82–3.31	0.2
**Fluid balance**	1.47	1.07–2.05	0.020
**Base Excess (BE)**	1.07	0.90–1.28	0.4
**Respiratory support after extubation**	1.17	0.83–1.65	0.4
**Mode of ventilation**	0.75	0.60–0.94	0.012
**Lactate**	1.18	0.96–1.53	0.14
**Urea**	0.94	0.72–1.24	0.7
**Creatinine**	1.48	0.78–3.12	0.3
**Haemoglobin**	0.82	0.69–0.97	0.022

**Table 2:** Demographic and clinical characteristics.

	Total	Overall	Success Extubation	Extubation Failure	*p*-Value ^1^
	N	824	593	231	
**Age in month**	824				0.1
**Median age (IQR)**		9 (2–48)	10 (2–54)	6 (2–37)	
**Sex**	823				0.7
Male		488 (59.3)	348 (58.8)	140 (60.6)	
Female		335 (40.7)	244 (41.2)	91 (39.4)	
**Source of admission**	823				<0.001
In hospital admission		585 (71.1)	409 (69.1)	176 (76.2)	
Out of hospital admission		238 (28.9)	183 (30.9)	55 (23.8)	
**Nutritional status**	820				0.029
Normal nutrition		656 (80.0)	481 (81.7)	175 (75.8)	
Undernutrition		158 (19.3)	102 (17.3)	56 (24.2)	
Overnutrition		6 (0.7)	6 (1.0)	0 (0.0)	
**Days of ventilation**	823				<0.001
**Median (IQR)**		3 (2–3)	2 (2–3)	3 (3–3)	
**Reason for EF**	824				
Upper airway obstruction		185 (22.5)	1 (0.2)	184 (79.7)	<0.001
Increased work of breath.		163 (19.8)	0 (0.0)	163 (70.6)	<0.001
Sepsis/septic shock		12 (1.5)	0 (0.0)	12 (5.2)	<0.001
Severe apnoea		25 (3.0)	0 (0.0)	25 (10.8)	<0.001
Neuromuscular weakness		17 (2.1)	0 (0.0)	17 (7.4)	<0.001
**Admission diagnosis**	824				
Respiratory pathology		354 (43.0)	244 (41.1)	110 (47.6)	0.10
Surgical and trauma		183 (22.2)	137 (23.1)	46 (19.9)	0.4
Sepsis and septic shock		118 (14.3)	81 (13.7)	37 (16.0)	0.4
Central nervous system		121 (14.7)	90 (15.2)	31 (13.4)	0.6
**Comorbidities**	824				
Congenital abnormalities		66 (8.0)	44 (7.4)	22 (9.5)	0.3
Renal disease		16 (1.9)	11 (1.9)	5 (2.2)	0.8
Oncology/haematology		34 (4.1)	22 (3.7)	12 (5.2)	0.3
Metabolic disease		12 (1.5)	9 (1.5)	3 (1.3)	>0.9
Cardiovascular disease		61 (7.4)	41 (6.9)	20 (8.7)	0.4
Central nervous disease		43 (5.2)	24 (4.0)	19 (8.2)	0.022
Others		152 (18.4)	101 (17.0)	51 (22.1)	0.11
No comorbidity		445 (54.0)	347 (58.5)	98 (42.4)	<0.001
**Planned extubation.**	824	824 (100.0)	593 (100.0)	231 (100.0)	
**PIM-3 mortality risk**	822	0.04 (0.02–0.07)	0.04 (0.02–0.06)	0.04 (0.02–0.09)	0.022
**Procalcitonin (PCT)**	822	0.80 (0.60–1.00)	0.80 (0.58–1.00)	0.84 (0.67–1.20)	<0.001

^1^ Wilcoxon rank sum test; Fisher’s Exact Test for Count Data; Fisher’s Exact Test for Count Data with simulated *p*-value (based on 2000 replicates).

## Data Availability

The original data presented in this study are available on reasonable request from the corresponding author. The data are not publicly available due to ethical restrictions.

## Supplementary Materials

The following supporting information can be downloaded at: https://www.mdpi.com/article/10.3390/reports9020169/s1.
