# Supplementary material for: Predictive Analysis of Extubation Failure in the Paediatric Intensive Care Unit in Bloemfontein, South Africa
Source: Reports (MDPI). 2026 May 28;9(2):169. doi: 10.3390/reports9020169 (PMC13306389; doi:10.3390/reports9020169)
Supplement: Supplementary file 1 [file reports-09-00169-s001.zip › reports-4181480-supplementary.pdf]

Model	Hyperparameters
XGBoost	Objective : binary:logistic<br>{ Objective : binary:logistic<br>"colsample_bytree": 0.3,<br>"gamma": 0.0,<br>"learning_rate": 0.005959467144720187,<br>"max_delta_step": 6,<br>"max_depth": 3,<br>"min_child_weight": 0.0,<br>"n_estimators": 506,<br>"reg_lambda": 2.3335425885702822e-05,<br>"scale_pos_weight": 1.0,<br>"subsample": 0.4,<br>}
Logistic Regression	Base Model
Support Vector Machine	{ "C": 0.9130497946548489,<br>"gamma": 0.019528196918645834,<br>"kernel": "rbf",<br>"degree": 2,<br>"coef0": 0.0,<br>}
Decision Tree	best_params = {<br>"ccp_alpha": 1e-6,<br>"class_weight": "balanced",<br>"criterion": "gini",<br>"max_depth": 26,<br>"max_leaf_nodes": 96,<br>"min_impurity_decrease": 0.0,<br>"min_samples_leaf": 1,<br>"min_samples_split": 38,<br>"min_weight_fraction_leaf": 0.0,<br>"splitter": "random",<br>"random_state": 42,<br>}
Random Forest	{<br>"bootstrap": True,<br>"class_weight": None,<br>"criterion": "entropy",<br>"max_depth": None,<br>"max_features": "sqrt",<br>"min_samples_leaf": 8,<br>"min_samples_split": 2,

	"n_estimators": 732,<br>"random_state": 42,<br>"n_jobs": -1,<br>}
Artificial Neural Network	{'epochs': 62, 'batch_size': 16, 'lr': 0.01, 'optimizer': 'nadam', 'activation': 'selu', 'neurons': 8, 'hidden_layers': 3, 'dropout': 0.0}<br><br>Output layer: Sigmoid
